# Evaluating CTLA-4 Antibody–Antigen Binding as a Model for Targeted Immunosuppressant Delivery in Hashimoto’s Thyroiditis

**DOI:** 10.17912/micropub.biology.001864

**Published:** 2025-12-15

**Authors:** Vinayan Tiruvellore, Mike Koegle

**Affiliations:** 1 College of the Canyons, Santa Clarita, California, United States; 2 Academy of the Canyons, Santa Clarita, California, United States

## Abstract

Autoimmune disorders such as Hashimoto’s thyroiditis lack curative treatments, only therapeutic agents that treat symptoms very broadly and dampen the entire immune system, including healthy cells. Cytotoxic T-Lymphocyte Antigen-4 (CTLA-4) is an immune checkpoint receptor expressed on T cells, and in Hashimoto’s thyroiditis the overexpression of CTLA-4 on dysfunctional immune cells is a key factor in disease progression. Here, we evaluated CTLA-4 antibody–antigen binding to specifically treat dysfunctional cells. Protein-protein docking identified Cluster 0 as the most stable pose (energy score –334), ChimeraX showed a buried surface area of 2,136 Å² and 1,023 hydrogen bonds, and in vitro assays confirmed binding.

**Figure 1. CTLA-4 antibody–antigen binding validation via computational docking and in vitro spectrophotometry:**
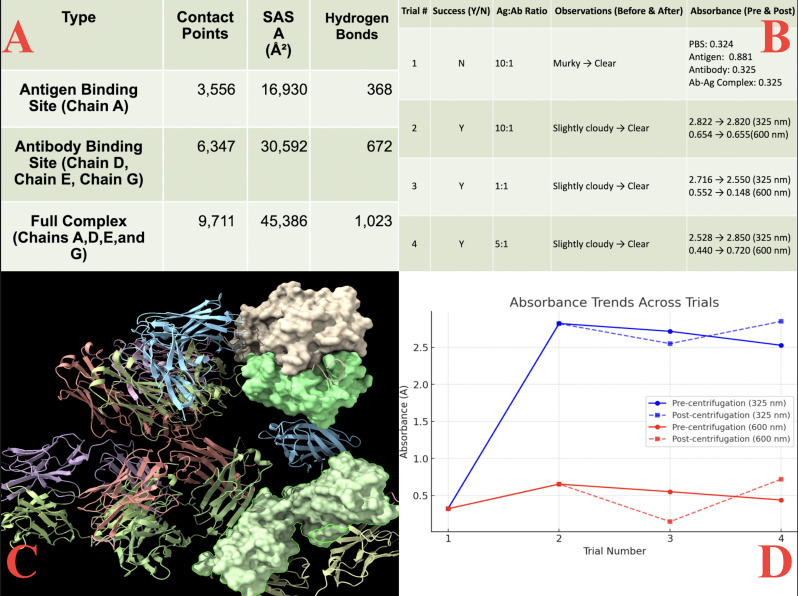
**(A)** Molecular interaction metrics calculated for the antibody–antigen docking complex. The ‘Antigen Binding Site (Chain A)’ row represents the CTLA-4 interface residues identified by the interaction analysis software. The ‘Antibody Binding Site (Chains D, E, and G)’ row corresponds to the antibody interface residues (heavy- and light-chain regions) as labeled in the analysis output. The ‘Full Complex (Chains A, D, E, and G)’ row reports the combined interface metrics for the entire antibody–antigen assembly. Values shown include the number of contact points, solvent-accessible surface area (SASA) involved in the interaction, and total hydrogen bonds formed within each region.” **(B)** Trial summary table including buffer conditions, centrifugation steps, and absorbance outcomes for antibody–antigen, antibody-only, antigen-only, and PBS controls.” **(C)** ChimeraX visualization of the final docked complex. The 8G8N heavy chain (chain A) is shown in light blue, the light chain (chain B) in pink, and the CTLA-4 antigen from 3OSK is shown as a biological homodimer (chains C and D) in green and beige. **(D)** Absorbance trends across trials. Curves are color-coded by wavelength: the 325 nm measurements are shown in blue and the 600 nm measurements are shown in red. Solid lines represent pre-centrifugation values, while dashed lines represent post-centrifugation values.

## Description

Cytotoxic T-Lymphocyte Antigen-4 (CTLA-4) is an immune checkpoint receptor expressed on the surface of T cells. It is responsible for inhibiting T cell activation and preventing excessive immune responses. This mechanism is essential for immune homeostasis and the prevention of autoimmunity (Wing et al., 2008). In autoimmune diseases such as Hashimoto’s thyroiditis, CTLA-4 dysfunction has been shown to be a key factor in the progression of these diseases. This indicates that dysfunctional immune cells have increased expression of CTLA-4 on their surface. This makes CTLA-4 a promising target for immunosuppressant delivery, offering a potential pathway for novel therapeutic interventions (Qureshi et al., 2011).

In developing therapeutics for this disease, the goal is to mitigate the immune-mediated destruction of the thyroid gland in Hashimoto’s thyroiditis while keeping healthy immune cells intact. Immunosuppressants affect the entire immune system, including healthy immune cells. Thus, it is necessary to discover a therapeutic treatment that exclusively targets overactive immune cells and prevent them from destroying the thyroid gland. To address this, we investigated whether a CTLA-4 monoclonal antibody can effectively bind the CTLA-4 antigen. Our hypothesis is that CTLA-4 monoclonal antibody will show a high binding affinity towards CTLA-4 antigen, substantial for therapeutic applications, and this binding can be quantified through *in silico* analysis and *in vitro* experimentation. The binding will be tested through protein-protein docking and protein visualization programs *in silico* , and a direct binding assay and spectrophotometer in vitro. If CTLA-4 antibody shows a significantly higher absorbance rate than controls, it indicates effective binding and can be considered in therapeutic applications to mitigate autoimmune response.

Docking analysis identified Cluster 0 as the most stable binding pose, with the lowest predicted *binding energy score* (–334, 57 poses), calculated by ClusPro as a weighted combination of van der Waals, electrostatic, and desolvation terms. ChimeraX visualization of this complex revealed a high buried surface area (amount of surface area actively involved in binding of the protein-protein complex) of 2,136 Å² and 1,023 hydrogen bonds, consistent with stable antibody–antigen interaction. As shown in Figure 1C, the 8G8N heavy and light chains (A and B) interact at the interface of the CTLA-4 dimer (chains C and D). In vitro assays further supported binding, as absorbance at 325 nm was significantly higher than in control conditions (PBS, antigen-only, or antibody-only solutions). Trials with centrifugation reduced background aggregation measured at 600 nm, with Trial 1 showing the strongest binding and later trials showing more consistent signals after aggregate removal. These results demonstrate effective antibody–antigen binding, supporting CTLA-4 as a potential model for targeted immunosuppressant delivery in Hashimoto’s thyroiditis. Refer to *Figure 1* for further numerical and observational data.

## Methods

For the in silico analysis, the 3D structures of the CTLA-4 antibody and antigen were retrieved from the Protein Data Bank (PDB) (Berman et al., 2000). The antibody structure was obtained from PDB ID 8G8N, which contains a Fab fragment composed of a heavy chain (chain A) and a light chain (chain B). The CTLA-4 antigen was obtained from PDB ID 3OSK, an apo homodimeric structure of the human CTLA-4 extracellular Ig-like domain; both monomers (chains C and D) were included in the docking analysis. These structures were processed through ClusPro, a protein–protein docking simulator (Kozakov et al., 2017), for docking analysis, and the most efficient binding model was subsequently examined using ChimeraX for visualization (Pettersen et al., 2020). Although 8G8N is a CTLA-4–specific therapeutic Fab, the deposited PDB structure does not contain CTLA-4. Because no experimental CTLA-4–8G8N co-crystal structure exists, the docking presented in this study represents a newly predicted CTLA-4–Fab interaction rather than a reconstruction of a previously solved antibody–antigen complex.

For the *in vitro* experiments, the antigen was first reconstituted by diluting two vials containing 50 μg of antigen each (100 μg total), in 500 μL of PBS to achieve a concentration of 100 μg/mL antigen solution, followed by thorough mixing with a pipette. Antibody dilution was performed by adding 980 μL of PBS to 20 μL of antibody stock to produce a working concentration of 10 μg/mL, generating a total of 1 mL of antibody solution. This solution was also mixed by pipetting. Binding assays were carried out by incubating 100 μL of the antigen solution (100 μg/mL) with 100 μL of the antibody solution (10 μg/mL) at room temperature for 30–60 minutes. Controls included 100 μL of PBS, plain antibody, and plain antigen.

To test reproducibility and diverse testing conditions, several trials were performed with varying concentration ratios and centrifugation. Centrifugation at 2,000 × *g* for 10 minutes was performed to observe if removal of aggregates influenced binding affinity. Trial 1 used an antigen-to-antibody ratio of 10:1. Trial 2 repeated the 10:1 ratio condition with an added wash step, including centrifugation at 2,000 × *g* for 10 minutes, to test removal of aggregates. Trial 3 matched the antigen-to-antibody ratio (10 μg/ml each), also with centrifugation as a wash step. Trial 4 tested a 1:2 antigen-to-antibody ratio, again followed by centrifugation. For each trial, absorbance was measured at both 325 nm to detect binding and 600 nm to detect additional aggregation.
